# "Detachment of the carinal hook following endobronchial intubation with a double lumen tube"

**DOI:** 10.1186/1471-2253-11-20

**Published:** 2011-10-28

**Authors:** Ana C Rocha, Mafalda G Martins, Luísa I Silva, José M Nunes

**Affiliations:** 1 Department of Anesthesiology, ULS de Castelo Branco, Portugal; 2 Department of Anesthesiology, Hospitais da Universidade de Coimbra, Portugal

## Abstract

**Background:**

Carinal hooks increases difficulty at endotracheal intubation. Amputation of the carinal hook during passage and malpositioning of the tube to the hook are some of the potential problems related with left-sided Carlens double lumen tube (DLT). This article reports an amputation of the hook during a difficult selective intubation and aimed at calling the attention to complications associated with DLTs and the importance of fiberoptic bronchoscopy.

**Case presentation:**

A 68 year-old woman was scheduled for right-sided thoracotomy in whom blind DLT insertion was performed. Narrowed trachea causes difficulty in rotating the DLT 90° counter-clockwise. After carinal hook was noticed upon visual inspection of the DLT, fiberoptic bronchoscopy was used to remove the missing part (with the use of forceps) from the right mainstem bronchus.

**Conclusion:**

Insertion of DLTs with carinal hook is associated with technical problems and potentially life-threatening hazards have discouraged their use. Fiberoptic evaluation and repositioning solves most of the problems. Although amputation of the carinal hook has not been previously reported, clinicians should be alert. This case report emphasizes the utility of the fiberoptic bronchoscopy in the operating theatre for placement, positioning and inspection of the carinal hook DLT.

## Background

Double-lumen tubes with fixed carinal hooks facilitated proper placement and minimized further tube advancement during positioning. However, potential problems and complications were associated with carinal hooks. These included a higher incidence of insertion difficulty, laryngeal trauma and amputation of the hook during placement [1,2]. Several methods for proper placement and positioning of DLTs are available in [3-6]. We describe a case of a carinal hook's amputation after blind insertion of left-sided polyvinylchloride Carlens DLT (SUMI^®^, Portex Inc., Mexico). The foreign body was removed from the right mainstem bronchial lumen with a fiberoptic-guided technique and, although a successful outcome was achieved, we failed the placement of DLT with fiberoptic bronchoscopy.

## Case Report

**Figure 1:**
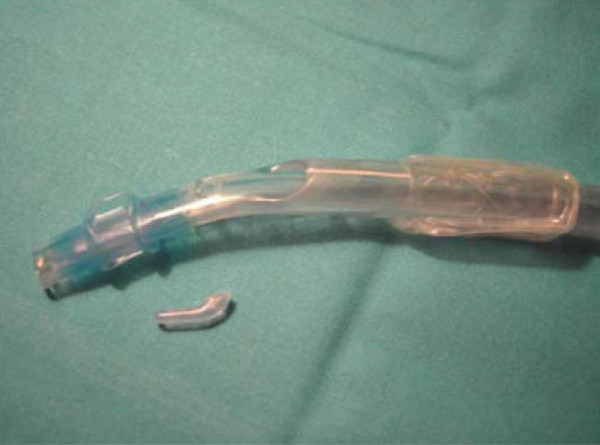
**Amputation of the carinal hook (arrows) in a left-sided polyvinylchloride Carlens DLT (SUMI^®^, Portex Inc., Mexico)**.

A 68 year-old woman (weight 74 kg, height 162 cm) with a pathological fracture of the fourth thoracic vertebrae (T4) was scheduled to undergo total removal of the vertebrae by a right transthoracic approach. Her past medical history included hypertension controlled with 20 mg enalapril once a day. Preoperative airway examination revealed a class II Mallampati with a normal mouth opening and normal dentition, a thyromental distance of 6.0 cm and limited neck extension. Paraplegia was present and no other abnormalities were detected on physical examination. All laboratory values, chest x-ray and 12-lead electrocardiogram were normal. The patient was premedicated with 10 mg diazepam on the night before surgery along with overnight fasting. After adequate preoxygenation, anesthesia was induced with 50 μg fentanyl, TIVA of propofol and remifentanil, and muscle relaxation was achieved with 50 mg rocuronium. Conventional direct laryngoscopy was performed and Cormack and Lehane grade was II (only the posterior extremity of the glottis visible). A 37F left-sided polyvinylchloride Carlens DLT (SUMI^®^, Portex Inc., Mexico) was placed using standard technique, although difficult to achieve because of a narrowed trachea. The patient's lung was ventilated through both lumens of the tube, with tracheal cuff inflated, but a resistance at manual ventilation was felt immediately after intubation. Chest auscultation revealed bilaterally equal and diminished breath sounds with pulse oximetry saturation decreasing from 99% to 96%. DLT was removed and before a second left endobronchial intubation attempt, examination of the DLT showed no carinal hook (Figure 1). An 8.0 mm ID single-lumen endotracheal tube was placed and then fiberoptic bronchoscopy was performed. An increased in pulse oximetry saturation (from 95% to 99%) with normal chest auscultation was noted. The fiberoptic examination showed the carinal hook located in the right mainstem bronchus, which was removed with the use of forceps. No evidence of a traumatized airway was noted. Two further attempts were made with another 37F Carlens DLT, by a different physician and with the aid of fiberoptic bronchoscopy (external diameter: 4.5 mm). However, DLT rotation failed because of a narrowed trachea. Smaller DLTs were not available. Thoracotomy was performed in left position with an 8.0 mm ID single-lumen endotracheal tube. The patient's hemodynamics was stable throughout this period. Her postoperative period was uneventful.

## Conclusions

Potential problems with carinal hook have been observed [1,2], however this is the first case reported of amputation of the carinal hook in a 37F left-sided polyvinylchloride Carlens DLT (SUMI^®^, Portex Inc., Mexico).

The exact mechanism leading to the amputate ion of the carinal hook is obscure. Rigid structures, narrow passages and manufacturing defect are possible [7].

In this case, desaturation and resistance to manual ventilation have alerted to a probably malpositioned DLT. In fact, tracheal narrowing was visualized by conventional laryngoscopy and insertion of the DLT occurred with some difficulties. The potential cause for the hook detachment was a narrowed trachea. Prompt action to remove the airway foreign body by bronchoscopy solved the patient's problem without adverse consequences. Although fiberoptic bronchoscopy is the accepted standard for appropriate positioning and confirmation of DLT [8], it was not performed in the first attempt. No double-lumen tube positioning method is fail-safe and unsuccessful replacement of another DLT with the aid of fiberoptic bronchoscopy occurred.

The present case has demonstrated that carinal hooks can be hazardous. It also highlights the importance of the availability of fiberoptic equipment in the operating theatre to promote proper DLT positioning and inspection of the carinal hook DLT, to reconfirm that no amputation had occurred.

Assuming a manufacturing defect of the 37F left-sided polyvinylchloride Carlens DLT (SUMI^®^, Portex Inc., Mexico.), until now no answer regarding the event was given by the DLT company.

## Consent

Written informed consent was obtained from the patient for publication of this case report. A copy of the written consent is available for review by the Editor-in-Chief of this journal.

## Competing interests

The authors declare that they have no competing interests.

## Authors' contributions

ACR collected data and drafted the manuscript. MGM collected the data and helped to write the manuscript. LIS and JMN reviewed the final manuscript. All authors read and approved the final manuscript.

## Pre-publication history

The pre-publication history for this paper can be accessed here:

http://www.biomedcentral.com/1471-2253/11/20/prepub
